# Sequence Does Not Matter: The Biomedical Applications of DNA-Based Coatings and Cores

**DOI:** 10.3390/ijms222312884

**Published:** 2021-11-28

**Authors:** Svetlana Batasheva, Rawil Fakhrullin

**Affiliations:** Institute of Fundamental Medicine and Biology, Kazan Federal University, Kreml Uramı 18, 420008 Kazan, Republic of Tatarstan, Russia; svbatasheva@gmail.com

**Keywords:** layer-by-layer assembly, polyelectrolyte multilayers, tissue scaffold, implant, flame retardancy, drug delivery, DNA-clay composites

## Abstract

Biomedical applications of DNA are diverse but are usually associated with specific recognition of target nucleotide sequences or proteins and with gene delivery for therapeutic or biotechnological purposes. However, other aspects of DNA functionalities, like its nontoxicity, biodegradability, polyelectrolyte nature, stability, thermo-responsivity and charge transfer ability that are rather independent of its sequence, have recently become highly appreciated in material science and biomedicine. Whereas the latest achievements in structural DNA nanotechnology associated with DNA sequence recognition and Watson–Crick base pairing between complementary nucleotides are regularly reviewed, the recent uses of DNA as a raw material in biomedicine have not been summarized. This review paper describes the main biomedical applications of DNA that do not involve any synthesis or extraction of oligo- or polynucleotides with specified sequences. These sequence-independent applications currently include some types of drug delivery systems, biocompatible coatings, fire retardant and antimicrobial coatings and biosensors. The reinforcement of DNA properties by DNA complexation with nanoparticles is also described as a field of further research.

## 1. Introduction

Admittedly, deoxyribonucleic acid (DNA) is the most important molecule on Earth, as it lies in the heart of life, containing all the necessary information for every organism development and functioning. In the DNA molecule, the genetic information is encoded as a sequence of nitrogenous bases attached to the phospho-deoxyribose backbone, with each DNA unit being called a nucleotide. The major DNA bases are adenine, guanine, cytosine and thymine, although some untraditional or modified bases can rarely be encountered. The first two bases are derivatives of purine and the second ones are pyrimidine derivatives. The specific interactions between the bases of adjacent DNA chains permit a complementary association of two single-stranded DNA (ssDNA) into one double-stranded DNA (dsDNA). The form of dsDNA is a double helix with major and minor grooves, in which hydrophobic planar bases are stacked inside the helix and the hydrophilic phospho-deoxyribose backbone is on the helix outside. Being heated up to about 80 °C, DNA undergoes a reversible denaturation that is separation of dsDNA into two DNA strands. If denaturation is followed by slow cooling, these ssDNA specifically recognize the base sequence of each other and self-assemble into original double-helical dsDNA.

The ability of DNA to encode genetic information envisions the main biomedical application of DNA as a drug used in gene therapy—a technology capable of editing the inherited genetic information of a human organism and thus improving the diseased condition [1]. Additionally, DNA can be delivered for vaccination purposes [2] or as a part of genetic engineering technology, which allows the production of new instruments for biomedical research (for instance, the fused fluorescent proteins) [3] or various biotechnology products [4]. In all these applications, the exact sequence of bases in DNA is of utmost importance. However, in recent years the distinct and relatively sequence-independent properties of the DNA molecule have found numerous applications in material science. The unique combination of such properties as nontoxicity, biodegradability, polyelectrolyte nature, stability, thermo-responsivity and charge transfer ability make DNA a useful resource material for fields ranging from optics and electronics to medicine and environmental protection. Apparently one of the most interesting DNA properties is its ability of specific recognition and self-assembly, which gave birth to DNA-tiles and DNA-origami technologies allowing the construction of complex pre-determined structures [5,6,7,8]. One of the most impressive sequence-specific DNA utilizations in bionanotechnology is aptamer-based recognition of proteins, tumor cells and low-molecular-weight hormones which is important for diagnostics and targeted drug delivery [9]. The specific Watson–Crick base pairing between complementary nucleotide sequences allowed the creation of smart DNA devices like DNA walkers, DNA tweezers, DNA robots and other DNA machines [10,11,12,13,14]. The DNA robots are so called because of their signal-processing ability and autonomous movements. The operating principles of DNA devices include the alternating hybridization and de-hybridization events between various complementary sequences (DNA strands displacement), which allows the device controllable “walking” [15]. In other designs, the transitions between states of a DNA device can be triggered by DNAzyme-catalyzed cleavage or external stimuli (pH, temperature, photoirradiation, electromagnetic field, etc.) [15]. Some DNA walkers can additionally sort, carry and drop useful cargoes, like proteins, small chemicals and metal nanoparticles [16]. DNA nanodevices are capable of revolutionizing the medical diagnostics, drug delivery and therapy, and the latest developments in this field were recently summarized in several excellent reviews [17,18,19,20].

**Scheme 1:**
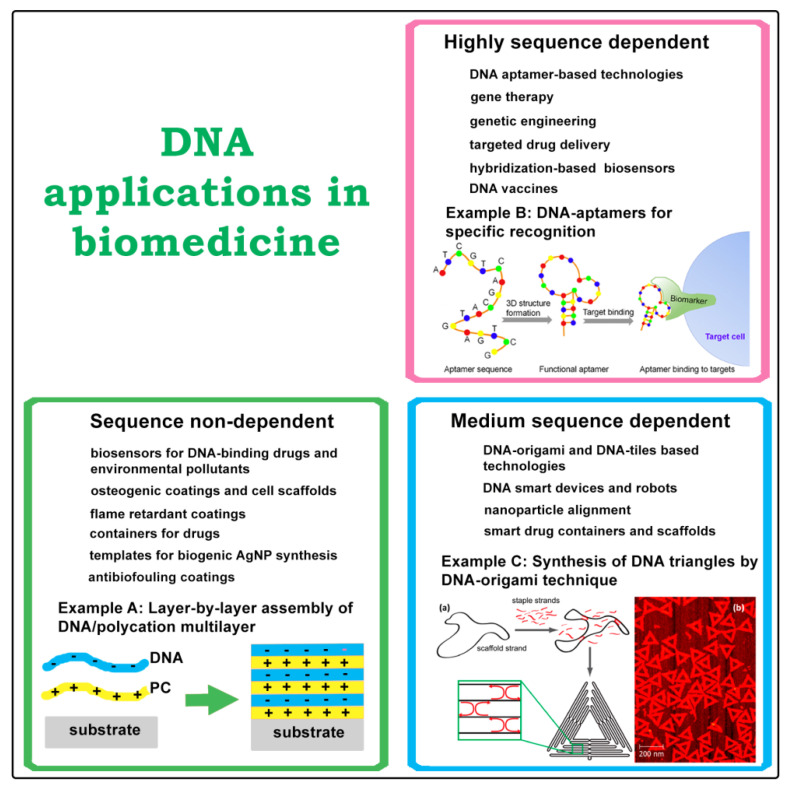
Main applications of DNA in biomedicine classified by their dependence on the specific nucleotide sequence. Example A: The technique of layer-by-layer assembly of a DNA/polycation (PC) multilayer from oppositely charged DNA and polycation molecules; Example B: Demonstration of specific recognition of a target cell receptor by an aptamer folded in the unique shape due to its specific nucleotide sequence. Reproduced from reference [24] under the CC BY license (http://creativecommons.org/licenses/by/4.0/, accessed on 25 November 2021) Example C: (**a**) Construction of DNA triangular nanostructures from a circular single-stranded scaffold DNA and short oligonucleotide staple sequences complementary to some regions of the scaffold DNA, (**b**) AFM image of triangular DNA structures. Reproduced from reference [25] under Creative Commons Attribution license (http://creativecommons.org/licenses/by/3.0/, accessed on 25 November 2021).

The current DNA-based designs are so numerous and versatile that they can hardly be comprehensively covered by one review. For simplicity, all current DNA-based technologies can roughly be divided into those not dependent on the exact polynucleotide sequence and those that are medium- or highly dependent on the specific sequence of DNA bases (Scheme 1). Usually, the reviews on DNA-based biomedical materials mainly focus on sequence-dependent applications of DNA [5,21], which look logical considering the unique ability of ssDNA to recognize a complementary nucleotide sequence with a striking specificity. However, sequence-independent biomedical DNA applications are also numerous, but have not been sufficiently reviewed so far. This review paper focuses on a sequence-independent group of DNA-based materials that are potentially useful for the biomedical field, leaving aside such intriguing DNA applications as DNA electronics and photonics, which were reviewed elsewhere [22,23].

## 2. DNA Based Drug Delivery Vehicles

The layer-by-layer (LBL) assembly technique was proposed in the 1990s as a way to form multilayered structures (polyelectrolyte multilayers) by sequential deposition of alternating oppositely charged polyelectrolytes [26]. Almost at once, DNA was tested as an anionic component of LBL-assembled multilayers [27]. Later, the spectrum of assembled materials was widened and now multilayers can be fabricated involving electrostatic, charge-transfer, hydrophobic, host–guest, coordination chemistry and biologically specific interactions, as well as covalent bonding, hydrogen bonding, stereocomplexation and surface sol–gel process [28], which allows obtaining tunable coatings and films.

Biodegradability, thermo-sensitivity and versatile binding abilities of DNA are attractive features for designing DNA-based drug delivery vehicles. DNA is capable of hosting other molecules by different modes, including intercalation between base pairs via hydrogen bonding and π–π stacking, geometrical compatibility with DNA major and minor grooves and electrostatic interactions with the DNA sugar-phosphate backbone [29]. Thermo-sensitivity of the DNA molecule allows the creation of thermo-switchable devices. This use of DNA is analogous to that of poly-N-isopropylacrylamide (PNIPAM) or other synthetic thermoresponsive polymers, but in contrast to them, DNA is more biocompatible [30].

**Figure 1:**
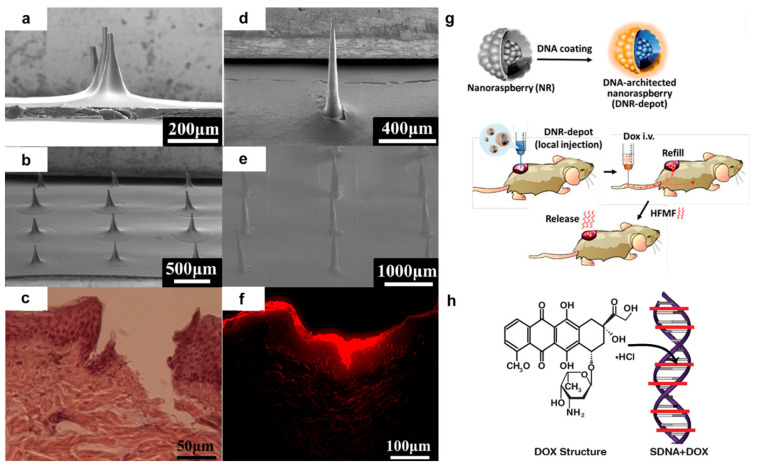
Scanning electron microscopy images of a single 300 µm-DNA microneedle (**a**), an array of 300 µm-DNA microneedles (**b**), a single 1000 µm-DNA microneedle (**c**), an array of 1000 µm-DNA microneedles (**d**), cross-section of a pork skin with a DNA microneedle-insertion site (**e**), model drug (rhodamine) distribution from the DNA-microneedle (**f**). Reproduced from [29] under Creative Commons CC BY license (http://creativecommons.org/licenses/by/4.0/, accessed on 25 November 2021). Copyright 2017 the authors; scheme of formation of DNA coated iron nanoparticles (nanorasberry) and their action as magnetoresponsive DOX depots (**g**). Reproduced with permission from [31]. Copyright 2020 Royal Society of Chemistry; schematic representation of DOX structure and its interaction with salmon DNA (**h**). Reproduced from [33] under Creative Commons CC BY license (http://creativecommons.org/licenses/by/4.0/, accessed on 25 November 2021). Copyright 2015 the authors.

The strong affinity between DNA and some small molecules allows using DNA as a reservoir of active agents for the purposes of controlled drug delivery. An injectable DNA-nanoraspberry depot (DNR-depot) serving as a sponge-like refilling reservoir for anticancer drug doxorubicin (Dox) (Figure 1h) was developed on the basis of porous iron oxide nanoparticles (nanoraspberries) covalently conjugated with double-stranded DNA [31]. This magneto-responsive DNR-depot was capable of local capture of intravenously injected anticancer drugs and their triggered release upon stimulation by a high-frequency magnetic field (Figure 1g). The DNR-depot was installed at the tumor site through intratumoral injection. After the DNR-depot had refilled itself with DOX, the tumor was subjected to a high-frequency magnetic field. In the process of magnetothermal conversion the DNR-depot was heated, which resulted in the temporary break of the DNA hydrogen bonds and drug release. The refilling/release cycle could be repeated several times to allow tumor suppression by increased local drug concentration. Administration of the depot and drug refilling demonstrated in vivo tumor suppression in mice. Additionally, the DNA coating was shown to contribute to a higher DNR-depot uptake by RG2 cells (a brain cancer cell line) in vitro, compared to the uncoated nanoparticles.

Good moulding ability of DNA allows obtaining structures of various sizes ranging from the nano- to micro-scale as well as patterned surfaces. Double stranded salmon DNA was applied to fabricate a microneedle system capable of transdermal drug delivery, in which DNA served as both a drug bearer and a structural material (Figure 1a–d) [29]. While the epidermis allows the permeation of only small molecules (less than 500 Da), microneedles create painless small holes in the skin, through which even molecules as large as proteins and nanoparticles can penetrate [32]. Using salmon DNA, mechanically strong and completely dissolvable microneedles were obtained, capable of delivering DNA-bound doxorubicine [29]. The microneedles were fabricated by casting DNA solution into poly-dimethylsiloxane moulds, obtained by a soft lithography technique. The successful transdermal delivery of drugs by the microneedles was proven by tracing rhodamine penetration through the stratum corneum of porcine skin [29] (Figure 1e,f). The microneedle fabrication technology is compatible with encapsulation of temperature-sensitive drugs as the moulding process takes place at ambient pressures and under 45 °C. When using DNA as a drug container, the optimal doping concentration of DOX was found to be 30 µM (per 0.5 wt.% DNA solution) in drug-doped DNA thin films [32].

Decomposable hollow capsules for the encapsulation and release of various substances were formed from DNA and a low molecular weight polyamine spermidine (SP) [34]. The DNA/SP multilayer coating was LBL-assembled on melamine formaldehyde core particles, which were then dissolved by acid treatment. The hollow DNA/SP capsules were sensitive to salt solutions and disassembled after exposure to sodium chloride solutions. However, the required NaCl concentration was rather high and thus further amendments of the multilayer assembly parameters are needed to allow its drug delivery applications.

The susceptibility of DNA to non-specific cleavage by reactive oxygen species (ROS) inspired the creation of H_2_O_2_-sensitive nanofilms consisting of DNA, hemin and poly(ethyleneimine) (PEI) [35]. Hemin is an iron porphyrin molecule, which served as a catalyst, producing more reactive oxygen species in its reaction with H_2_O_2_. Hemin was either non-covalently adsorbed on the (PEI/DNA)_5_ nanofilm or conjugated with the PEI constituent. Both nanofilms were destroyed by H_2_O_2_ addition, but the nanofilm containing hemin-appended poly(ethyleneimine) was more H_2_O_2_-sensitive and decomposed at H_2_O_2_ concentrations as low as 10 mM.

This design was later developed into a glucose-sensitive nanofilm, composed of five bilayers of LBL-assembled hemin-appended poly(ethyleneimine) (H-PEI) and glucose oxidase-loaded DNA [36]. When glucose oxidase reacts with glucose, gluconic acid and H_2_O_2_ are produced, and H_2_O_2_ further reacts with hemin to generate ROS which cleave DNA and promote the nanofilm degradation. The LbL films were slowly decomposed under physiological conditions at a low glucose concentration (1–10 mM), with the rate of decomposition increasing with glucose concentration, which suggests the use of these glucose-responsive nanofilms in prototypes of insulin delivering systems.

Further development of DNA drug containers may benefit from the technology of DNA amphiphiles as well as researches primarily aimed at gene therapy such as DNA compaction with polycations [37,38]. DNA amphiphile is a hydrophilic DNA containing a covalently connected hydrophobic moiety, which allows formation of DNA-based micelles and other complex structures. The presence of a hydrophobic segment facilitates the interaction of DNA amphiphiles with cell membranes [39].

Recently DNA microcapsules were developed on the basis of “Janus-like” DNA surfactants spontaneously generated from dsDNA and amine-functionalized polyhedral oligomeric silsesquioxane (POSS-NH_2_) at the oil–water interface [40]. POSS-NH_2_ as a surfactant assembled at the oil–water interface and electrostatically interacted with DNA solubilized in water phase, which resulted in formation of an interfacial elastic film. By changing the concentrations and ratios of POSS-NH_2_ and DNA, and the ionic strength or pH of the aqueous solution the structure and properties of the interfacial assembly were tuned. As a result, the assembly assumed a solid-like, elastomer-like or liquid-like physical state. Using the microfluidic or emulsification technique, a one-step formation of DNA capsules was achieved, which were stable after air drying and capable of encapsulating various water-soluble molecules, including bovine serum albumin.

## 3. Cytocompatible and Antibiofouling DNA Coatings

**Figure 2:**
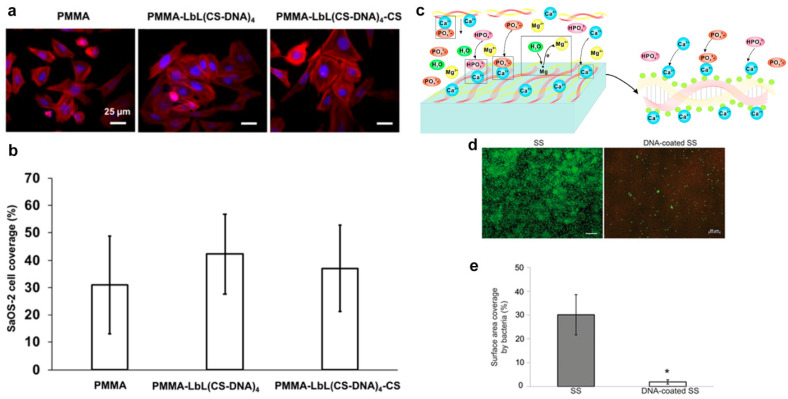
Fluorescent microscopy images of SaOS-2 cells grown on uncoated poly(methyl methacrylate (PMMA) surfaces or PMMA surfaces coated with chitosan/DNA multilayers (PMMA-(CS-DNA)_4_ and PMMA-(CS-DNA)4-CS) for 48 h (**a**) and the surface coverage by the cells after 48 h growth (**b**). Reproduced from [58] under the Creative Commons Attribution (CC BY) license (https://creativecommons.org/licenses/by/4.0/, accessed on 25 November 2021; the mechanism of DNA-induced Ca-P deposition on the surface of Mg alloy (**c**). Reproduced without modifications from [50] under the CC BY-NC-ND license (http://creativecommons.org/licenses/by-nc-nd/4.0/, accessed on 25 November 2021); the images of SYTO-9-stained bacterial biofilms adhered to stainless steel surfaces after 48 incubation obtained by fluorescent microscopy (scale bar is 20 µm) (**d**) and the surface coverage by the bacteria after 48 h growth (**e**). A statistically significant decrease in bacterial adhesion to DNA-coated surface compared to the uncoated one is indicated by an asterisk. Reproduced with permission from [59]. Copyright 2019 Taylor & Francis.

The beneficial properties of eukaryotic DNA as a component of biomedical materials include its non- or low-immunogenicity [41], and thus high biocompatibility, ability to incorporate various compounds [42] and high phosphate content. Additionally, the DNA-based coatings are rather stable as complexation of DNA with cationic polyelectrolytes protects DNA from degradation by nucleases [43,44]. Phosphate groups of the DNA backbone can bind Ca^2+^ ions and promote the deposition of Ca-P complexes (Figure 2c), which made DNA an attractive component for producing coatings for bone implants [45,46,47,48,49,50,51]. The complexation of DNA with various cytocompatible polycations, such as chitosan, polyamino acids and protamine, DNA/poly-d-lysine or DNA/poly(allylamine hydrochloride) was tested [44,45,52,53,54,55,56]. DNA/poly-d-lysine or DNA/poly(allylamine hydrochloride) LBL assembled multilayers enhanced calcium phosphate deposition from simulated body fluid (SBF) [45]. Additionally, the degradation of DNA can release phosphate ions to the bone microenvironment and promote osteogenesis, upregulating the expression and activation of sodium-dependent phosphate co-transporter by phosphate [57]. When DNA/protamine complex was implanted through an incision into soft tissues of the skin of Sprague–Dawley rats, the complex completely degraded and disappeared within 10 days [56]. The degradation rate of the DNA containing complexes depends on the DNA molecular weight [44]. 

Magnesium (Mg) alloys are currently regarded as a material of choice for making biodegradable bone implants [60] because Mg is involved in the regulation of osteogenesis [61] and the Mg alloy elastic modulus is close to that of natural bones [62]. The Mg-based implant should provide mechanical support and promote tissue healing, and eventually degrade in the organism when it is no longer needed [63]. However, too rapid degradation of Mg alloys is unfavourable and research efforts are focused on designing surface modifications capable of preventing the preterm degradation of Mg-based implants [64,65,66,67,68]. 

Using the LBL technique, DNA was alternated with polyvinylpyrrolidone (PVP) (a cationic polyelectrolyte) to obtain a (PVP/DNA)_20_ multilayer on a Mg-containing AZ31 alloy and to induce the formation of a terminating Ca-P coating by a hydrothermal deposition process [69]. The LbL-assembled DNA and PVP promoted the mineralisation of Ca-P, producing a Ca-P coating with good corrosion resistance, tested by soaking the sample into Hank’s balanced salt solution for 250 h. The positive effect of DNA on Ca-P mineralization was greater when it was used as a part of the LBL assembly than alone, which was evidenced by a lower corrosion resistance of Ca-P coatings induced by DNA compared to that of (PVP/DNA)_20_. To further increase the corrosion resistance of DNA-coated Mg-based implants as well as impart them with antibacterial effect, a cationic PVP could be replaced with chitosan (CS) and an underlying layer of Mg(OH)_2_ could be added to the coating [68]. To assess the cytocompatibility of (CS/DNA)_5_Mg(OH)_2_ coatings mouse MC3T3-E1 pre-osteoblast cells were grown for 1, 3 and 5 days in α-MEM growth media supplemented with 10%, 25%, 50%, 75% and 100% of the coating extract. Cell viability measured by a colorimetric MTT (3-(4,5-dimethylthiazol-2-yl)−2, 5-diphenyltetrazolium bromide) test demonstrated a 10% decrease in viability for 100% extracts and no statistically significant influence on cell viability for lower percentage extracts [68].

Osteogenic potential of DNA can also be useful for fabrication of coatings on dental titanium (Ti) implants. Protamine, a group of arginine-rich proteins, has antibacterial and antifungal effects [56,70], which makes its use in coatings as a DNA counter-ion beneficial for implant resistance to bacterial biofilm formation. The DNA/protamine implanted into rat calvarial defects enhanced bone regeneration [71,72] and promoted the expression of osteogenic genes (RUNX-2, alkaline phosphatase, osteopontin and osteocalcin) in cultured mesenchymal-like cells [72]. DNA/protamine complex increased the alkaline phosphatase activities of cultured osteoblast-like cells [73]. Protamine was first tightly bound on the Ti implant surface using a tresyl chloride-activated method and then DNA of 300 bp or 7000 bp was electrostatically deposited and alternated with the following protamine layers until five protamine/DNA bilayers were assembled, terminating in DNA [74]. Wettability and surface roughness of coated Ti discs were different depending on the DNA length, and a more hydrophilic and uniform coating was formed by 7000 bp DNA/protamine multilayers. The deposition of apatite was faster on the DNA/protamine-coated Ti implants than on the non-treated Ti implants, and the rate of bone healing process after implant positioning in the extracted sockets of rat molars in animal experiments was faster with DNA/protamine coatings. A higher osteointegration of titanium implants coated with ssDNA/protamine or dsDNA/protamine multilayers was also demonstrated in vivo in a rat model [75]. A first molar tooth of a rat was extracted and the screw-type implant was placed into the root socket. The subcutaneous injection of calcein at 2 or 5 weeks after the surgery allowed monitoring of new bone formation in the implantation site by the fluorescence of calcein-calcium complex. The authors concluded that although both ssDNA and dsDNA promoted early apatite deposition and new bone formation, the use of ssDNA is less expensive, but in contrast to dsDNA it does not allow inclusion of cytokines or antibiotics [75]. The complex of DNA and protamine becomes a paste after being kneaded with water, but the physical properties of the conventionally prepared paste do not allow its easy use in everyday dental practice [76]. Another paste consisting of DNA and gelatine was proposed, which had better bone formation ability than that of the DNA/protamine paste in the extracted sockets of rat maxillary molars [76]. To prepare DNA/gelatin paste 50 mg of gelatin was mixed with 400 mL of water, and then 50 mg of DNA was added. The authors suggested that a higher rate of new-bone formation by the DNA/gelatine paste compared to the DNA/protamine one was associated with its faster degradation in the implantation site.

The careful choice of polycations paired to negatively charged DNA to make coatings for tissue engineering scaffolds and implants is of high importance because of the significant toxicity of some synthetic polycations to mammalian cells [77]. To overcome this problem, DNA can be combined with natural polycations such as chitosan, which is a highly biocompatible linear cationic polysaccharide, obtained from crustaceans and currently widely applied in various biomedical designs [78,79]. However, the mechanical properties of pure DNA/polycation multilayers can be rather poor, and underlayers of alternating highly negatively and positively charged synthetic polyelectrolytes can be used to solve this problem. Thus, three bilayers of PEI-DNA polyelectrolytes formed structurally weak, hydrated films with a high viscous modulus, while when highly charged polyelectrolytes PEI and polystyrene sulfonate (PSS) were used in the initial three bilayers, more compact and rigid films were formed [58].

The insoluble transparent DNA films can be obtained by curing DNA with UV radiation, inducing the formation of cross-linking –O-P-O– bonds [80]. An aqueous solution of salmon DNA (5 wt.%) was casted on a Teflon-coated glass plate, water was slowly evaporated at 40 °C and the film was UV-cured for 1 min. As a result, an insoluble film was formed, permeable to oxygen, carbon dioxide and water vapour, which was capable of supporting the growth of rat chondrogenic ATDC5 cells and exerting a healing effect when applied to wounded rat skin [80].

The ability of DNA to intercalate or electrostatically associate various substances can be used for functionalization of DNA coatings with growth factors and other regulatory molecules. DNA/poly-d-lysine (PDL) or DNA/poly(allylamine hydrochloride) (PAH) coatings were loaded with osteoinductive factor bone morphogenetic protein 2 (BMP-2), which remained biologically active without affecting cell proliferation of bone marrow-derived osteoblast-like cells [46]. Recombinant human BMP-2 was electrostatically incorporated as a deep or superficial layer (or both) into the DNA/PDL or DNA/PAH coatings on titanium substrates. All functionalized DNA coatings showed an initial bulk release of the protein within 24 h followed by a sustained release for 8 weeks, irrespective of the cationic counterpart of DNA in the multilayered coating. An accelerated mineralization (calcium deposition) by osteoblast-like cells was observed on the coatings that contained BMP-2 in the superficial layer.

In the other study, vascular endothelial growth factor (VEGF) was deposited on the top of a coating, consisting of five bilayers of PDL and DNA on a glass substrate [47]. Positively charged VEGF was loaded at 25 or 250 ng by electrostatic adsorption onto the negatively charged DNA top-layer. These VEGF-functionalized DNA-based coatings increased proliferation and migration of endothelial cells (human umbilical vein endothelial cells) in vitro. Being implanted in the backs of Wistar rats in an in vivo animal model for 1–3 weeks, the coatings significantly enhanced vascularization of the peri-implant area without causing any negative effects in the experimental animals. No statistically significant differences between two VEGF concentrations were observed.

As a charged polymeric molecule, DNA is an effective stabilizer for nanoparticles (NP), capable of both steric and electrostatic stabilization. The additional steric stabilization allows the nanoparticles to remain suspended even in the high ionic strength solutions where electrostatic stabilization is neutralized [81]. Different methods were proposed for nanoparticle modification with DNA, such as nanoparticle wrapping in dry DNA with ball milling [82,83] or adsorption of DNA on nanoparticles in the presence of divalent cations [84]. The high negative charge of DNA-modified NP allows their use as building blocks in the evaporation-driven self-assembly of nanoparticles into complex ordered structures [84] because the dispersion stability is a critical factor for a successful self-assembly process [85]. Concentric ring patterns obtained from DNA-modified clay nanotubes using the so called “coffee ring effect” turned out to be very durable and washing-resistant, while the structures made from the nanotubes stabilized with synthetic polyelectrolytes were easily broken and washed away with water [84]. These washing-resistant complex patterns can be used for formation of nanostructured tissue scaffolds, cell capturing or in filtering devices [86]. 

Interestingly, although the inclusion of DNA into coatings do not prevent attachment of mammalian cells and even improves the biocompatibility of various materials (see above), the use of the same molecule is effective for fabrication of coatings, preventing bacterial adhesion. Almost all known microorganisms, upon reaching a certain colony density, form biofilms - complex communities of microorganisms embedded in the extracellular matrix, consisting primarily of biopolymers. The formation of microbial biofilms leads to a decrease in the efficiency of various industrial devices, and is also the cause of persistent infections, as the bacteria embedded in biofilms may be protected from antimicrobial agents [87]. The biofilms contribute to the development of bacterial resistance by allowing non-dividing and therefore antibiotic-insensitive bacterial subpopulations to survive among antibiotic-susceptible organisms [88]. Moreover, when the biofilm is destroyed, planktonic forms of microorganisms with increased virulence can be released [89]. Thus, prevention of biofilm formation, rather than destruction of biofilms, should be the preferred approach to control microbial contaminations and here surface coatings that prevent attachment of microorganism could be of great help. 

DNA-based coatings can prevent adhesion of bacterial cells and biofilm formation [58,59,90], although it cannot be concluded at present what are the key factors determining DNA antibiofouling properties. They are attributed to the negative charge of DNA molecules resulting in repulsive interactions with negatively charged surfaces of bacteria [59,90] and topography of the coatings [59]. Reduced protein adsorption due to high hydration of DNA layers was also proposed as an explanation of antibiofouling effects of DNA [58]. It was found that the size of surface-attached DNA molecules is of great importance for the coating antibacterial effect, although the underlying mechanism is still elusive [90]. Salmon sperm DNA of three different molecular weights (<500 bp, ∼5–15 kbp and >20 kbp) were adsorbed or covalently attached to Si wafers and allylamine plasma polymer (AAMpp)-coated Si wafers, and the antibacterial effect of the resulting surfaces to *Pseudomonas aeruginosa* was tested. The highest bacteria-repelling effect was observed for all surfaces modified with small sized DNA, while large DNA (<500 bp) did not reduce the attachment of bacteria to Si wafers compared to control [90]. 

PEI/DNA LBL coating on stainless steel reduced by 93% the formation of the microbial biofilm when incubated statically or in flow with unchlorinated tap water, and reduced the attachment of the human pathogens *Staphylococcus aureus*, *Staphylococcus epidermidis* and *P. aeruginosa* to glass surfaces (Figure 2d,e) [59]. Six double layers of PEI/DNA terminating in a DNA layer were formed by sequential immersion in corresponding polyelectrolyte solutions (0.5 mg/mL) for 5 min intermittent by water rinsing. Reduced biofilm formation on DNA-coated surfaces was a result of prevented bacterial attachment rather than their growth, as most of the bacteria adhered to the DNA-coated glass surface remained viable. The DNA coatings also had an anti-scaling effect, both at room and elevated temperatures, preventing CaCO_3_ deposition on the coated surfaces in analogy to phosphate-based additives.

Chitosan-DNA (CS/DNA) polyelectrolyte coatings were created by successive deposition of four CS/DNA bilayers on PMMA and Ti surfaces precoated with three PSS/PEI bilayers [58]. The obtained coatings were resistant to biofouling and biofilm formation by *S. aureus* and *S. epidermidis* pathogens. The antifouling (bacteria-repelling) effect of DNA was retained even when it was covered by chitosan as the uppermost layer, while the sole CS coating on a PMMA surface was inefficient. The adhesion and spreading of osteoblast-like SaOS-2 cells after 48 h on the DNA-multilayer coated PMMA was similar to that on an uncoated PMMA surface (Figure 2a,b), suggesting high biocompatibility of the obtained coatings and their possible use for fabrication of bacteria-resistant Ti implants. 

Thus, the use of DNA and a proper cationic counterpart allows formation of composites having dual properties of cytocompatibility and bacteria resistivity, which is highly promising for fabrication of biomedical materials. Moreover, such methods of physical DNA organization as molecular combing or DNA deposition on a rotating substrate [91] potentially allow fabrication of tissue scaffolds with complex structured surfaces.

## 4. Antibacterial and Flame-Retardant Coatings for Fabrics Based on DNA

At present, the most widely used fire retardants include brominated, chlorinated and organo-phosphorus compounds, which are harmful for environment and human health [92]. Thus, new approaches are sought for producing non-toxic and eco-friendly halogen-free fire retardants. One of these approaches consists in the use of intumescent fire-retardant systems [93]. Being heated, intumescent fire retardants form a swollen multicellular char that protects the underlying material from fire, inhibiting the heat and oxygen transfer [93]. Intumescent flame retardant behaviour is associated with the chemical structure including a carbon source, an acid donor and a blowing agent, which allow interrupting the self-sustained combustion. Intumescent coatings are capable of providing fire resistance to different substrates, like metals, textiles, plastics and foams [94]. 

The chemical composition and structure of DNA makes it an intumescent fire retardant, as it contains phosphate groups (the phosphoric acid donors), deoxyribose sugars as a carbon source and blowing agents together with purine and pyrimidine bases which may release ammonia under heating. Upon heating to about 200 °C, DNA is decomposed, producing a protective char layer and foamed thermally insulating material that is thermally stable up to 600 °C [94]. 

DNA is very effective in imparting cellulosic fabrics with flame retardant properties [95,96]. Anticipation of cotton cellulose dehydration by DNA at lower times and temperatures promotes the char formation instead of the release of volatile compounds [94]. Initially, the addition of 19 wt.% of low molecular size DNA from herring sperm was used [95], but later research on the effect of different DNA add-ons (5, 10 and 19 wt.%) indicated that 10 wt.% was the minimum value necessary to provide the cotton with self-extinction behaviour in horizontal flame spread tests [96]. It was found that the DNA-coated cotton fabrics did not burn at all after two applications of a methane flame for 3 s, and did not ignite when exposed to an irradiative heat flux of 35 kW/m^2^ [96]. The general flame-retardant features of DNA depend on its molecular size, pH of the DNA aqueous solution at the cotton impregnation process and number of impregnations [97]. The coatings (8 wt.%) containing short DNA (100–200 bp) conferred better fire protection on the fabric substrate than medium (400–800 bp) and high (2000–10,000 bp) sized DNA, as was indicated by horizontal flame spread and cone calorimetry tests. The better fire-retardant behaviour of the low molecular weight DNA was attributed to its greater penetration into the cotton microfibrillar structures and to its higher thermal stability in air. The comparison of efficiency of a single cotton treatment at a higher DNA concentration or multiple treatments at a lower concentration demonstrated that repeated impregnations are substantially more effective for protecting the cotton fabrics than the single one, despite the same level of final DNA add-on [97]. 

The pre-treatment of cotton fabrics with citric acid to increase free carboxylic group content and hence DNA absorption significantly improved the flame resistance of the DNA-treated cotton [98]. The length of DNA coated fabrics damaged by flames was only 29 mm compared to the control length of 350 mm, and the cotton fabric after DNA finishing could stop flame propagation (self-extinguish). The adsorption of herring sperm DNA on cotton fabric was fast, with the sorption reaching saturation within 30 s. However, after five washings, the damaged length of cotton fabrics became similar to the untreated fabrics suggesting that the DNA finishing could be used for non-durable (disposable) flame-retardant products [98].

DNA was combined with chitosan for layer by layer assembly of multilayer coatings containing 5, 10 and 20 DNA/chitosan bilayers on cotton fabrics with a total dry mass of 2.5, 7 and 14 wt.%, respectively [99]. Despite being very thin, the 20 bi-layer coatings were capable of providing the cotton self-extinction during horizontal flammability tests and reducing the heat release rate by 40% in cone calorimetry tests. Later studies confirmed the flame-retardant efficacy of DNA/chitosan composite coatings, but also demonstrated their antimicrobial activity [100]. However, in randomly mixed DNA/chitosan composites the antimicrobial activity of chitosan was compromised due to the involvement of its critically important protonated amino groups in interaction with DNA, while the layer by layer assembled chitosan-DNA UV-cured coatings provided water resistance, self-extinction in flammability tests and a good antimicrobial activity to cotton fabrics [100].

Recently, a superhydrophobic and flame-retardant coating on cotton fabric was obtained by sequential immersion of the fabric into the solutions of calf thymus DNA, AgNO_3_ and octadecyltriethoxysilane (ODTS) [101]. The resulting coating was thermally and chemically stable and retained its superhydropohibic properties for at least four washing cycles in detergent and six cycles in water.

**Figure 3:**
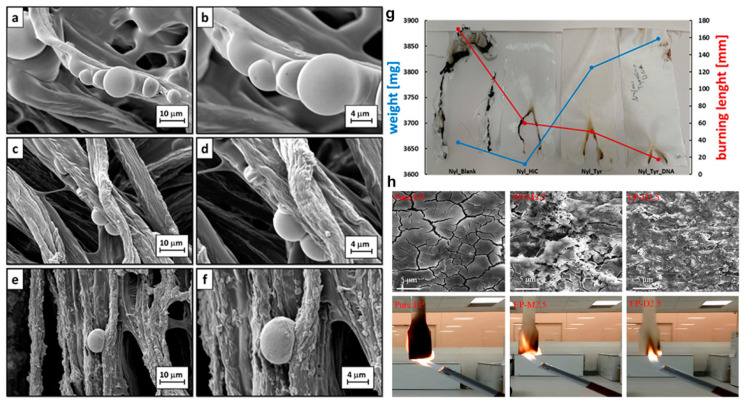
SEM images of cotton fabrics, coated with 10 polydiallyldimethylammonium chloride (PDAC)/DNA bilayers without (**a**,**b**) and with addition of 0.1% (**c**,**d**) and 1.0% (**e**,**f**) of hydrotalcite, after its exposure to a flame retardant test. Reproduced from [102] under the Creative Commons Attribution (CC BY) license (https://creativecommons.org/licenses/by/4.0/, accessed on 25 November 2021); pure nylon (Nyl) and nylon acitvated with cutinase (Nil_HiC) followed by treatments with tyrosine (Nyl_Tyr) and DNA (Nyl_Tyr_DNA) after a flammability test (**g**). The lines indicate the changes in the remaining fabric weight (blue) and burning length (red) depending on the treatment. Reproduced from [92] under the terms of the Creative Commons Attribution License (CC BY). Copyright 2019 the authors. SEM images of char and 10th second of flame retardance testing of pure epoxy resin (Pure EP) and epoxy resin containing 2.5 wt.% of Nanomer I.2E organoclay (EP-M2.5) or DNA-modified montmorillonite (EP-D25) (**h**). Reproduced from [103] Creative Commons Attribution license (https://creativecommons.org/licenses/by/4.0/, accessed on 25 November 2021). Copyrigh 2016 the authors.

To enhance the thermal resistance of DNA coatings ceramic (or clay) nanoparticles are sometimes added. The polydiallyldimethylammonium chloride (PDAC)/DNA LBL coatings were formed on cotton fibres and then treated with hydrotalcite nanoparticles (0.1 and 1 wt.%) that diffused into the coatings and improved their fire-retardant properties [102]. The LBL coating comprising five PDAC/DNA bilayers reduced the cotton burning rate, while ten bilayers were capable of self-extinguishing behavior. Upon coating heating, the degradation of the DNA component began, resulting in the formation of thermally stable protective charred structures and coating bubbling (Figure 3a). The expanded coating acted as a barrier, slowing down the release of combustible volatiles from the degrading cotton fabric and the heat transmission from the flame to the cotton, thus resulting in a flame-retardant effect. The treatment with hydrotalcite nanoparticles at 0.1 wt.% concentration improved the fireproofing properties of the coating, but high nanoparticle concentration (1.0%) was detrimental for flame retardant performance of the LBL coating because of particle aggregation that hindered coating expansion (Figure 3a–f).

The flame retardant and washing fastness of cotton fabrics was significantly increased by applying a hybrid coating made of TiO_2_ NPs and DNA [104]. The interaction between TiO_2_ NP and DNA was driven by electrostatic forces, and 4 or 8 layered composite coatings were assembled on the cotton fabrics due to the cross-linking action of TiO_2_ that strongly interacted both with DNA and the cotton substrate. The TiO_2_/DNA coatings showed good flame-retardant properties, increasing the total burning time (even achieving self-extinction), while decreasing the burning rate, and the resistance to the flame propagation improved with the increasing number of deposited layers. The homogeneous protective coating around the fibres preserved the original texture of the fabrics during combustion. The authors proposed to use this approach of an easy DNA immobilization on cellulose substrate not only for creating durable flame-retardant coatings but also for other applications in the field of biotechnology and nano-medicine.

Flame retardant DNA-based coatings are also compatible with synthetic polymers, like poly(ethylene terephthalate) (PET) and nylon-6, widely applied in furniture, wall insulation, curtains, flooring and clothing [92]. For PET and nylon modification with DNA, anchor groups (i.e., carboxyl groups) were first introduced into the synthetic fabrics via limited surface hydrolysis with *Humicola insolens* cutinase (HiC). Then, low molecular weight DNA (20–30 bp) from salmon sperm was immobilized on the activated polymers by using three different coupling systems: EDC/NHS, dopamine and tyrosine. Dopamine and L-tyrosine are natural low molecular weight compounds, which makes them eco-friendly cross-linking agents. The covalent dopamine and tyrosine-linked coatings demonstrated greater washing durability compared to the coatings obtained by EDC/NHS cross-linking method, but the dopamine coating gave a dark brownish colour to both textiles. Thus, the best cross-linker for DNA immobilization on PET and nylon-6 was tyrosine. The tyrosine/DNA coated PET samples had a decreased burning rate and showed more intact fibres after combustion, while control PET fabrics were melted. Tyrosine/DNA seemed to be even better treatment for nylon fabric, because the flame was self-extinguished nearly immediately after the flame was applied to the fabric (Figure 3g). The DNA coating also preserved the structure of underlying nylon fibres, which were protected from the attack of heat.

DNA, either applied as a bulk melt-blended flame retardant [105] or a coating [106], turned out to exert a thermal shielding effect on ethylene vinyl acetate (EVA) copolymer containing 18 mass% of vinyl acetate, a thermoplastic material used in electrical cables and biomedical devices. However, DNA was more effective when it was deposited on EVA surface, instead of being added to the bulk, because a flame retardant loaded in the bulk had to diffuse toward the surface in order to perform its action [106]. Unlike DNA in bulk, DNA in coating exhibited exceptional thermal shielding properties and was capable of protecting the underlying material from a butane/propane torch during three consecutive applications for 5 s. To prevent DNA washing out from the flame retardant coating it can be embedded into UV-cured polymer matrix as it was demonstrated for a UV-cured acrylic resin (Bisphenol A hydroxyl ethyl diacrylate) and DNA (10 and 15 wt.%) coating on EVA copolymer [107]. The high aspect ratio of DNA facilitated its interaction with the polymer matrix, resulting in good DNA distribution and reinforcing effect.

Recently, DNA-modified natural clays were tested as dopants for increasing the flame retardancy of polymer materials [103,108]. Two clays, differing in structure, sodium cloisite and sepiolite, were modified with low molecular weight DNA (less than 50 bp) from herring sperm and then melt compounded with EVA copolymer (19% of vinyl acetate) at 10 wt.% loading [108]. The clay filles (irrespective of their DNA modification) exerted only a slight effect on the EVA copolymer flammability, but lowered the heat release rate, the peak of heat release rate, the total smoke production and the specific extinction area. The authors suggested, that performance of DNA-modified fillers was probably limited by their partial aggregation (especially in the case of sepiolite) evidenced by SEM. 

The other research group modified montmorillonite (MMT) clay with waste DNA from fishing industry in order to increase the clay interactions with epoxy resin and improve its thermo-physical properties [103]. The maximum amount of 72 mg DNA per gram of clay was intercalated within clay layers at pH = 2. After the modification, the previously hydrophilic clay became more hydrophobic and concentrated in an organic phase (chloroform) instead of water phase. DNA-MMT incorporated into the epoxy resin (2.5 wt.%) significantly enhanced the resin flame retardant and mechanical properties, namely tensile strength, tensile modulus and fracture toughness. It demonstrated better flame-retardant behaviour than a commercial clay-based dopant Nanomer I.2E added at the same concentration (Figure 3h). In addition to its intrinsic flame-retardant activity, DNA contributed to uniform clay dispersion and clay-resin interactions, further enhancing mechanical and thermal properties of the obtained material. This study also confirmed the remarkable flame-retardant properties of DNA, because addition of pure DNA (2.5 wt.%) resulted in the highest improvement of the epoxy resin flammability. Unfortunately, pure DNA can deteriorate mechanical performance of epoxy polymers, but its grafting to clay can compensate the reduction in mechanical properties. While higher amounts of DNA-modified nanofillers are expected to be more efficient in terms of the composite thermal behaviour, too large nanofiller loadings may exert negative influence on the mechanical performance of epoxy composites, and thus only a small add-on of DNA modified clay was recommended by the authors [103].

Recently, DNA was tested as an agent for reinforcing the mechanical properties of poly(lactic acid) (PLLA) bioplastics [109]. DNA/PLLA composites exhibited slightly better thermal and mechanical properties than pure PLLA, but were less efficient than PLLA/collagen composites.

**Figure 4:**
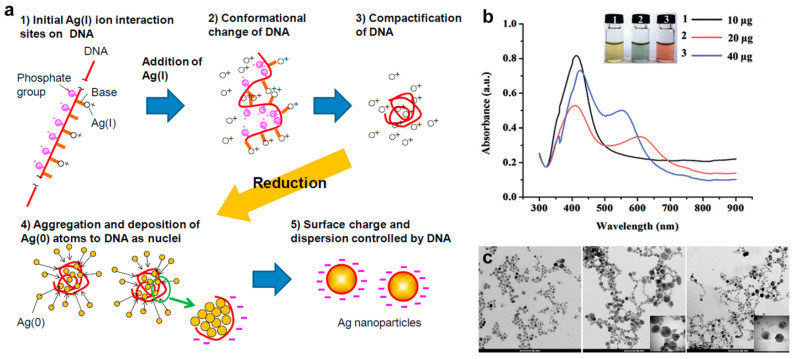
A schematic representation of DNA-promoted synthesis of AgNP (**a**). Reproduced with permission from [111] under the Creative Commons Attribution (CC BY) license (https://creativecommons.org/licenses/by/4.0/, accessed on 25 November 2021. UV-Vis spectra of AgNP (**b**) and TEM images (scale bar is 50 nm) (**c**) of AgNP obtained under blue light and different DNA content (left–10 µg, middle–20 µg, right–40 µg). Reproduced with permission from [114]. Copyright 2018 Taylor & Francis.

There are fewer studies devoted to fabrication of DNA-based antibacterial coatings for fabrics than fire-retardant ones and in all of the studies the antibacterial effects are attributed to silver nanoparticles, whose antimicrobial activity is well known [110]. The coating produced from DNA-covered silver nanoparticles can impart fabrics with antibacterial properties [111]. By using DNA as a template and a stabilizer (Figure 4a), a large amount of spherical AgNP with an average diameter less than 10 nm was produced [112]. Ag(I) ions were bound by DNA nucleotide bases and reduced by treatment with NaBH_4_ solution. The resulting nanoparticles were highly negatively charged due to the adsorption of a thin DNA layer and thus stable in water. The electrostatic deposition of DNA/AgNP on the surface of cotton fabrics treated with cationic diallyl(3-chloro-2-hydroxypropyl)amine hydrochloride-diallyldimethylammonium chloride copolymer resulted in formation of an antibacterial coating efficient against *Escherichia coli* bacteria [111]. Recently, a bacterial genomic DNA was used as a stabilizing agent for the light-activated synthesis of AgNPs [113]. NPs with an average size of 61.36±10.15 nm were formed, which exhibited antibacterial activities against *E. coli* and *S. aureus* bacteria. In another study, plasmid DNA and irradiation with light emitting diodes guided the formation of differently shaped AgNP [114]. All-spherical yellow NP, synthesized under blue light irradiation and low DNA content, demonstrated higher antibacterial effect than anisotropic AgNP synthesized at larger DNA contents (Figure 4b,c). Noteworthy, the plasmid DNA templated organization of inorganic and organic building blocks was first shown in 1990s and included the guided assembly of CdS nanoparticles and fullerenes [115].

In all the above studies the antibacterial effects of the DNA-coated AgNP were assigned to the AgNP core rather than the presence of DNA. However, as it was mentioned earlier, DNA coatings have their own antibacterial activity [90], but an assessment of the separate roles of AgNP and DNA in antimicrobial effects was not provided. It can be expected that the range of DNA-modified materials will be extended in the future and more studies on natural (for example, wool) as well as synthetic fabrics will appear. The use of DNA composites with other clay species also looks promising, considering the binding ability of clays towards natural human and animal hair [116,117].

## 5. DNA as a Part of Electrochemical Biosensors

DNA electrochemical sensors for detection of environmental toxicants and drugs are based on the electrochemical signals of the DNA bases after the interaction with analyte molecules [118]. Among the DNA bases, guanine is the most susceptible to oxidation [119], although all four DNA bases are electrochemically active [120]. The mechanism of DNA-mediated electrochemistry is still not fully understood and thus is actively studied [121,122]. As a most common mechanism, it is believed that electrons as well as electron holes are transported through the DNA π-stack [123].

The charge transport (CT) along the stacked DNA base pairs strongly depends on the integrity of the DNA and any substances or other impacts perturbing the base pair stacking interrupt the DNA charge transport [122]. In DNA electrochemical sensors registering the presence of some pollutants and drugs, dsDNA extracted from various sources is usually used and the nucleotide sequence of this DNA is not of high importance as opposed to the ssDNA-based sensors intended for specific recognition of target oligonucleotides by DNA-hybridization events [122].

The process of DNA monolayer formation is a major step affecting the stability, reproducibility and sensitivity of the DNA sensor [120,122]. There are two general methods for DNA immobilization on the electrode surface: A covalent bonding or an electrostatic deposition using the LBL technique. Thiol-modified DNA and gold nanoparticles are often used for dsDNA immobilisation on the electrode surface, because of the high affinity of gold to thiol groups and its conductivity [124,125,126], while unmodified ssDNA can be directly adsorbed onto gold surfaces [122]. The covalent DNA immobilization is more robust, but it is also more expensive. The density of the DNA monolayer on the electrode surface can be regulated by the addition of MgCl_2_ during the deposition process to minimize electrostatic repulsion between adjacent DNA duplexes [122].

Electrochemical DNA biosensors can be applied for detection of DNA damage induced by hydroxyl radicals and screening of different compounds for their antioxidant activity [127,128]. The oxidative damage of the DNA structure (base and sugar lesions (including the loss of bases) and strand breaks) results in the increased charge transfer resistance (Rct) assessed by electrochemical impedance spectroscopy or voltammetry [129,130,131].

To detect the DNA damage induced by the Fenton reaction and to test the antioxidant capacity of deferoxamine an electrochemical biosensor based on DNA/Au nanoparticles modified screen printed gold electrode (DNA/AuNPs/SPGE) was developed [132]. The charge transfer resistance dramatically increased after the sensor had been immersed into the damaging solution, while no increase in Ret was observed if deferoxamine was present in the damaging solution, indicating the antioxidant activity of deferoxamine.

The ability of DNA to bind some drug molecules (first of all, anti-cancer preparations) can be used for their detection with DNA-based electrochemical biosensors. A number of electrochemical sensors based on hybrids of DNA and redox-active electropolymerization products, like polyaniline, polypyrrole, polythiophene, polyphenazines and their derivatives, were proposed to register the interaction of DNA with DNA intercalating drugs [133,134,135,136,137]. There are different ways for DNA inclusion into the electrode coating, containing electropolymerization products: electrostatic adsorption together with or without the use of other polyelectrolytes [136] or DNA entrapment during the electropolymerization stage [138]. An electrochemical DNA-sensor based on glassy carbon electrode modified with electropolymerized neutral red (NR) and polycarboxylated thiacax[4]arene was developed for detection of anthracycline medicines (doxorubicin, daunorubicin and idarubicin). The sensor exhibited high sensitivity and detected the concentrations as low as 0.05 nM of doxorubicin, 0.1 nM of daunorubicin and 0.5 nM of idarubicin [136].

Recently, the inclusion of a macrocycle polyaminothiacalix[4]arene to biosensors based on DNA complexes with polyaniline and poly-neutral red was proposed as a possible way of increasing the sensitivity of the sensors towards doxorubicin detection, as the macrocycle forms complexes with DNA and partially shields the negative charge of its phosphate groups [139].

Another design of a biosensor capable of detecting DNA-drug interactions, oxidative DNA damage and probing antioxidant compounds was based on a surface plasmon resonance (SPR) chip, coated with DNA/PAA multilayers [140]. The concentration range of doxorubicin detection by the SPR sensor was 1 × 10^−12^ to 1 × 10^−7^ M with the limit of detection of 7 × 10^−13^ M, which is sufficient for doxorubicin monitoring in urine and other biological fluids. The SPR sensor registered DNA oxidation by a mixture of hydrogen peroxide and Cu^2+^. The DNA damage suppressed the SPR signal, because of partial destruction of PAA/DNA complexes and leaching of DNA fragments from the surface of the SPR chip. The anti- and pro-oxidant activities of low and high concentrations of ascorbic acid, respectively, could also be demonstrated by changes in SPR angle shift, although the SPR sensor could not be used for quantification of ascorbic acid because of a narrow dynamic range.

Aflatoxin B1, a food contaminant produced by *Aspergillus* fungi found on grain crops was assessed using a DNA-based biosensor, consisting of the carbon paste electrode modified with N-butylpyridinium hexafluorophosphate ionic liquid and DNA [141]. The biosensor response was linear in the range from 8.00 × 10^−8^ to 5.91 × 10^−7^ M with a limit of detection of 2.00 × 10^−8^, which allows its use for determination of low concentrations of aflatoxin B1.

The antibiotics ciprofloxacin (from the group of quinolones) and sulfadiazine (from the group of sulfonamides) were successfully detected with multi-walled carbon nanotubes (MWCNT)/DNA modified glassy carbon electrodes [142,143]. The electrode modification was achieved by direct immobilization of DNA adsorbed on MWCNT. A DNA biosensor for antidepressants promethazine and chloropromazine from the group of phenothiazine drugs was obtained by co-immobilisation of DNA and gold nanoparticles on a gold electrode [144].

In addition to drug-DNA intercalation studies, DNA-based electrochemical sensors are useful for studying the mechanisms of DNA recognition by proteins, which is important for new drug development, considering the involvement of DNA–protein interactions in the regulation of gene expression [145].

The additional materials are employed to improve the performance of the DNA-based sensors, because the direct immobilization of DNA on electrodes is unstable and the electrochemical signals of thus immobilized DNA bases are generally weak [146,147,148]. The inclusion of nano-thin exfoliated layered double hydroxide (LDH) sheets can be regarded as a way to facilitate the electron transfer between DNA and the electrode [149]. Positively charged exfoliated Ni-Al-LDH sheets were included as a layer between negatively charged DNA and Nafion layers deposited on the PEI-modified surface of a glass electrode using the LBL technique to make an electrochemical sensor of an antidiabetic drug phenformin hydrochloride [120]. The influence of the DNA concentration, the number of DNA layers as well as the use of dsDNA or ssDNA on the sensor performance were tested and it was found that a monolayer of dsDNA deposited from 10mg/mL solution was optimal. The interaction of phenformin hydrochloride with dsDNA was stronger than that with ssDNA due to dual interaction mechanism including both electrostatic attraction and intercalation between the DNA chains [120]. The electrochemical sensor was stable and showed signal linearity from 1.0 × 10^−5^ mol·L^−1^ to 1.0 × 10^−3^ mol·L^−1^ with a detection limit of 3.4 × 10^−6^ mol·L^−1^, and this linearity range was significantly larger than those of other phenformin hydrochloride detection methods, including HPLC and capillary electrophoresis [120].

**Figure 5:**
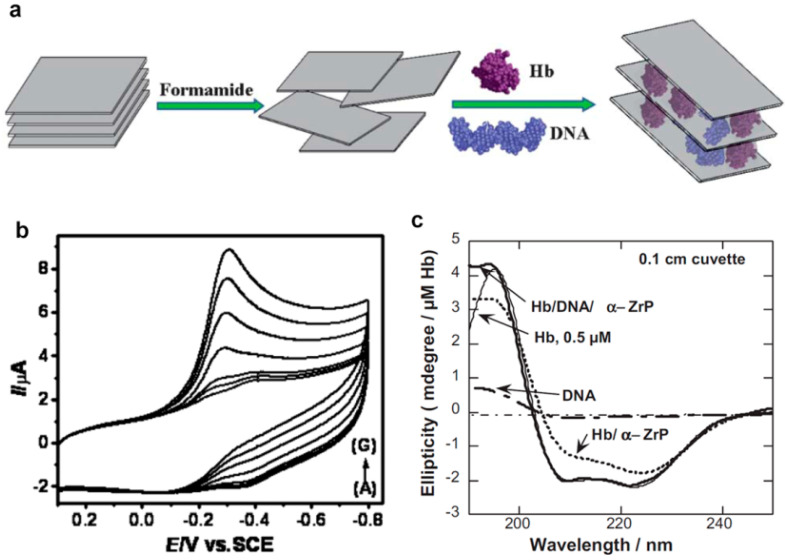
A scheme of co-intercalation of hemoglobin and DNA into the LDH interlayers (**a**) and cyclic voltammograms of Hb/DNA/LDH electrode incubated in a buffer with increasing H_2_O_2_ concentrations from (A) to (G), where (A) = 0, (B) = 0.97, (C) = 9.71, (D) = 48.52, (E) = 97.05, (F) = 145.58, (G) = 194.1 µM H_2_O_2_ (**b**) Reproduced with permission from [150] Copyright 2013 Royal Society of Chemistry. Circular dichroism spectra of Hb (thin line), DNA (dashed line) and Hb bound to α-ZrP in the presence (thick line) or absence of (dotted line) DNA (**c**). Reproduced with permission from [151]. Copyright 2006 John Wiley and Sons.

DNA can facilitate the intercalation of other biomolecules into the interlayer spaces of LDH and thus make possible their protection from adverse environmental factors. An electrochemical H_2_O_2_- and NO_2_^−^-responsive biosensor was obtained by deposition of a hemoglobin/DNA/Ni-Al-LDH composite onto a glassy carbon electrode (Figure 5a,b) [150]. The Hb/DNA/LDH composite was fabricated by a delamination-reassembly process at pH 9.0 (i.e., above the isoelectric points of DNA (pH 4.0–4.5) and Hb (pH 6.8–7.0)) to benefit from electrostatic interactions between the negatively charged biomolecules and the positively charged clay nanosheets (Figure 5a). The XRD study demonstrated a significant increase in the interlayer spacing of Hb/DNA/LDH composites (4.0 nm) in comparison with both pristine LDH (0.88 nm) and LDN/Hb hybrid (2.93 nm). The biosensor exhibited a linear and reproducible electrocatalytic response to H_2_O_2_ and NO_2_^−^ in the range of 4.85 × 10^−7^ to 1.94 × 10^−4^ M and 2.5 × 10^−7^ to 3.0 × 10^−5^ M, respectively. Intercalation into clay particles protected the Hb from high temperatures (85 °C) and the presence of an organic solvent, acetonitrile, thus providing stable functioning of the biosensor under harsh external conditions.

This study also demonstrated that co-intercalation with DNA significantly enhanced the catalytic activity of the LDH-immobilised Hb [150]. This result is in accordance with an earlier research, where co-intercalation of DNA and Hb into the galleries of layered α-zirconium phosphate increased the activity of bound Hb due to the DNA-promoted improvement of the a-helical content of the bound Hb to the level of the free protein (Figure 5c) [151].

As far as we know, in all dsDNA-based electrochemical biosensors developed up to date, only eukaryotic (or synthetic) DNA was used. However, the use of archaeal or bacterial genomic DNA might be interesting, considering the higher content of electrochemically active G-C base pairs in some prokaryotic genomes [152]. In addition to its role as a component of biosensors for screening environmental pollutants, DNA can be used for their capturing [153], but this DNA application remains understudied.

## 6. The Limitations, Challenges and Future Prospects of Raw DNA Applications in Biomedicine

The biomedical applications of raw DNA have some limitations. Despite a rather high stability of DNA to physical impacts compared to other biomolecules, it can be degraded by extra- or intracellular nucleases when exposed to microorganisms and human cells, which decreases the durability of DNA-based materials. If DNA is used as a drug container, the stepwise degradation of exogenous DNA structures and the fate of DNA degradation products in the organism must be studied. There were no special studies on the destiny of raw DNA-based drug delivery systems in mammals, although some information is available from studies on DNA-based vehicles produced by the structural DNA nanotechnology [154]. The unmodified DNA nanostructures were stable in serum within 4–25 h [155]. The stability of DNA systems is dependent on the number of single strand DNA segments, because these are more prone to degradation by nucleases [156]. Various chemical modifications can shield the DNA nanostructures from hydrolysis, with the nanostructure topology and high packing density of DNA molecules also exerting some protective effects [157]. As the exogenous DNA differs from the self one it can potentially invoke a strong immune response [158] and thus immunogenicity must be carefully tested for every DNA-based system to be introduced into a human body. As partially dissociated DNA nanodevices could potentially elicit stronger immune responses and toxicities, the reinforcement of DNA structures by covalent linkages could be used as a way to protect them from degradation [158]. In contrast to aptamer-based drug delivery vehicles, the targeting ability of raw DNA-based delivery systems is limited and thus the systems proposed so far had to be directly injected into the target site [30,31]. However, the additional modification of DNA layers with targeting molecules like proteins or peptides is possible to overcome this limitation. The results of the studies on the mechanism of cell penetration of DNA-based vehicles and their intracellular fate are still contradictory [155] and thus require further research. The data inconsistency is probably explained by imperfect methods currently used for tracking the movement of DNA-based systems and more precise data will be obtained if better analytical techniques are developed in the future [155].

Other perspective avenues for future research on biomedical materials based on raw DNA can be outlined. The surprising ability of DNA to improve the structure of some proteins intercalated into layered nanoclays may possibly find applications in protein drug delivery. However, this phenomenon was as yet demonstrated only for the family of heme proteins, which have unusually high DNA binding affinity [159], and thus it requires further research. The ability of DNA to stabilize emulsions [40] and nanoparticle dispersions [82,83,84] can find wider applications in pharmaceutical and cosmetic formulations, provided the immunogenicity aspect is properly addressed.

Currently most studies use DNA extracted from eukaryotic organisms. However, eukaryotic and prokaryotic DNAs have different ratios of nucleotide species as well as different immunological properties, which indicates their possible different behaviour in practical applications. Moreover, a very interesting problem that awaits further research is the mechanism of size-dependent antibacterial effect of DNA [90].

As the biomedical DNA applications often imply the use of sterile materials, the development of sterilization procedures is of high importance. The currently used methods for sterilization of DNA-based materials were recently reviewed [21]. Thanks to ever-evolving methods of DNA extraction and purification, the price is no longer a serious limitation to the technologies based on raw DNA. DNA can now be obtained in large amounts from sources like fish waste [153], brewer’s yeast and vegetable scraps [160], which makes industrial-scale production of DNA-materials a reality and justifies the development of new DNA-based designs.

The antibacterial and mechanical properties of raw DNA-based systems can benefit from combinations with nanoclays and metal nanoparticles, and more studies are expected in this field in the future.

## 7. Conclusions

The sequence-independent DNA properties advantageous for its use in the biomedical field can be summarized as follows. DNA is a natural polyelectrolyte molecule, which can replace the synthetic polyelectrolytes because of its lower toxicity and biodegradability. Thermo-sensitivity and mouldability of DNA allows creation of thermo-switchable and nano-architectured drug delivery systems. The responsivity of the DNA structure to binding of other molecules and DNA charge transfer ability make it a useful component of biosensors for screening environmental pollutants, drugs and antioxidant agents. Being an intumescent fire retardant, DNA is an eco-friendly and non-toxic alternative to currently used fire retardants. DNA that originates from eukaryotic organisms is cytocompatible with mammalian cells but cytophobic to bacteria, although the latter effect depends on the DNA length. Certainly, this list of beneficial properties is incomplete and will be extended as our knowledge about DNA grows, further widening the application range of raw DNA material in biomedicine.

## Data Availability

Not applicable.
